# Recent Progress in *Shigella* and *Burkholderia pseudomallei* Vaccines

**DOI:** 10.3390/pathogens10111353

**Published:** 2021-10-20

**Authors:** Itziar Chapartegui-González, Sarah Bowser, Alfredo G. Torres, Nittaya Khakhum

**Affiliations:** 1 Department of Microbiology and Immunology, University of Texas Medical Branch, Galveston, TX 77550, USA; itchapar@utmb.edu (I.C.-G.); saainswo@utmb.edu (S.B.); 2 Department of Pathology, University of Texas Medical Branch, Galveston, TX 77550, USA

**Keywords:** *Burkholderia pseudomallei*, *Shigella*, vaccines, live attenuated, glycoconjugate vaccines, subunit vaccines

## Abstract

Significant advancement has been made in the development of vaccines against bacterial pathogens. However, several roadblocks have been found during the evaluation of vaccines against intracellular bacterial pathogens. Therefore, new lessons could be learned from different vaccines developed against unrelated intracellular pathogens. Bacillary dysentery and melioidosis are important causes of morbidity and mortality in developing nations, which are caused by the intracellular bacteria *Shigella* and *Burkholderia pseudomallei*, respectively. Although the mechanisms of bacterial infection, dissemination, and route of infection do not provide clues about the commonalities of the pathogenic infectious processes of these bacteria, a wide variety of vaccine platforms recently evaluated suggest that in addition to the stimulation of antibodies, identifying protective antigens and inducing T cell responses are some additional required elements to induce effective protection. In this review, we perform a comparative evaluation of recent candidate vaccines used to combat these two infectious agents, emphasizing the common strategies that can help investigators advance effective and protective vaccines to clinical trials.

## 1. Introduction

One of the current threats in public health according to the World Health Organization (WHO) is the rise of multidrug-resistant bacteria (https://www.who.int/news-room/fact-sheets/detail/antimicrobial-resistance, accessed on 1 August 2021). That challenge is even higher when trying to combat intracellular bacterial pathogens for which there are no approved vaccines. In this review, we will compare the virulence paths of two different pathogens, the diarrhea-causing enterobacteria *Shigella* and the causative agent of melioidosis, *Burkholderia pseudomallei* (*Bpm*), and discuss the approaches and most recent advances into vaccine development to combat both infections.

### 1.1. Pathogens and Diseases Incidence

Although at first glance, these pathogens seem unrelated, and both are responsible for quite distinct diseases in different endemic regions, they share some virulence features. *Burkholderia* and *Shigella* are Gram-negative bacilli with sophisticated intracellular lifestyles that cause damage to the host cells and tissues, resulting in human disease.

*B. pseudomallei* is the agent causative of melioidosis, which is a mainly human disease that has also been seen in other mammals (e.g., sheep) [1]. *B. pseudomallei* is classified as Tier 1 Select Agent by the U.S. Centers for Disease Control (CDC) because of its biothreat potential [2]. On the other hand, *Shigella* is responsible for bacillary dysentery, which is a disease commonly known as shigellosis in humans and other primates [3].

While *Shigella*-caused dysentery has an estimated annual worldwide burden of 80–165 million cases [4], *B. pseudomallei* causes an estimate of 165,000 melioidosis cases annually [5]. Shigellosis is responsible for up to 600,000 deaths per year with 55,000 of these in children under 5 years of age. In contrast, around 89,000 melioidosis cases are fatal, which represents more than half of the total diagnosed cases per year. *Burkholderia* is endemic in Northern Australia and Southeast Asia, while reports are increasing in tropical areas of Asia, Africa, as well as Central and South America [5]. *Shigella* is a food-borne pathogen found worldwide, but the main endemic areas are tropical and subtropical regions in sub-Saharan Africa and Southeast Asia [6].

*Shigella* is a very contagious, low-dose pathogen, with a high person-to-person transmission rate—a spreading process that is very rare during *Burkholderia* infections. Among the total shigellosis cases, 20 to 119 million are linked to food-borne ingestion [4]. In contrast, *Bpm* is known to use the inhalational, percutaneous, and gastrointestinal routes to enter the host and disseminate to target organs. Interestingly, stool samples from people in endemic areas suffering with melioidosis have shown that *B. pseudomallei* can cause gut colonization, highlighting the ingestion of contaminated food or water as a likely infectious route [7,8].

The genus *Shigella* is formed by four species or subgroups (*S. flexneri*, *S. sonnei*, *S. dysenteriae* and *S. boydii*) and a total of 43 serotypes, which represents the variability of the lipopolysaccharide (LPS) O antigen—the major antigenic target recognized by the host immune system [3]. Among all the serotypes, *S. flexneri* represents more than half of total worldwide cases. In the case of *B. pseudomallei*, two main phylogenetic linages have been identified [9]: one that is predominant in Northern Australia and one that circulates in Southeast Asia. However, other new linages have been described in south Asia, Africa, and the Americas [10,11].

### 1.2. Pathogenesis and Life Cycle

Both *Shigella* and *B. pseudomallei* are facultative intracellular pathogens, who despite the different infectious routes share similarities in their life cycle. As with any other intracellular pathogen, both bacteria need to invade their target cell once they adhere to the epithelium (e.g., intestinal, respiratory, or cutaneous). While *B. pseudomallei* uses different infectious routes, the most studied one is the invasion of epithelial cells in the respiratory tract. On the other hand, *Shigella* targets intestinal M (microfold) cells in the colonic epithelial barrier.

*B. pseudomallei* invasion in non-phagocytic epithelial cells is mediated by adhesins (BoaA, BoaB), type IV pili (PilA), and type I fimbriae (FimA), which is associated with intestinal colonization [1,12,13]. *Shigella* is a non-flagellated bacillus requiring the help of the host to reach the epithelial surface, and upon contact, it elicits filopodium-mediated motility dependent on the type 3 secretion system (T3SS). The early *Shigella* invasion steps are still poorly understood, but the role of the T3SS transcription factors VirF and VirG as well as the translocator proteins IpaB and IpaD has been described [3]. The T3SS is a syringe-like mechanism employed by Gram-negative pathogens to translocate effectors inside the target cells through the plasma membranes. In the case of *B. pseudomallei*, it is known that isolates harbor three T3SS clusters in their genome, and the expression of the T3SS-3 (*bsa* locus), homologous to the *S. flexneri* T3SS, is triggered after host cell contact and has been associated with both non-phagocytic cells invasion and endocytic vacuole escape [14].

Once *Shigella* and *B. pseudomallei* have invaded epithelial cells, they use the T3SS to escape from the endocytic vacuoles, reaching the cytosol where both pathogens can actively replicate. Cytosolic replication of *Shigella* is also mediated by T3SS through the injection of a second wave of effectors regulated by MxiE, which can repress the host inflammatory response and ensure the favorable conditions for the bacteria in the cytosolic niche [3]. In *B. pseudomallei*, several structural proteins (BsaQ, BsaZ), as well as effectors (BopE, BopA) and translocator proteins (BipB, BipD), have been described, but these proteins have a role more associated with cellular invasion than inflammation control. Both pathogens use these mechanisms and effectors to subvert the cytoskeleton, using them to manipulate actin filaments, allowing intracellular motility [15,16].

Both bacteria also possess type 6 secretion systems (T6SS), which are virulence mechanisms that function by delivering effector proteins directly into eukaryotic and prokaryotic target cells, with distinct differences between the two systems [3,14]. *Shigella* uses its T6SS to compete with host microbiota before reaching the mucus layer in the colonic epithelium [3]. In contrast, T6SS is one of the most important features in *B. pseudomallei* pathogenesis, with some of its functions mediating cell-to-cell spread and the formation of multinucleated giant cells (MNGCs). In the *B. pseudomallei* genome, six different T6SS gene clusters have been identified, while only T6SS-1 has a role in intracellular survival [17]. The proteins that compose the *B. pseudomallei* T6SS assemble into three different subcomplexes: the tubular system in the cytoplasm with the contractile TssB and TssC proteins and an inner tube formed by Hcp1 that ends in a sharp structure formed by VgrG; an envelope spanning membrane complex formed by TssM, TssL, and TssJ; and a base plate that anchors the tube and sheath to the membrane [17].

These pathogens are not only able to invade and replicate into non-phagocytic cells, but they can survive inside macrophages. Another common characteristic in the life cycle of both pathogens is that they trigger a caspase-1 dependent cell death mechanism known as pyroptosis [3,12]. This process requires T3SS participation, and once the intracellular bacteria are released, they can disseminate to distal organs (*B. pseudomallei*) or invade the replicative niche in the colonic epithelium (*Shigella*).

### 1.3. Vaccines and Animal Models

Despite the incidence or mortality of these pathogens, there is still no approved vaccines for human use. Interestingly, the vaccine platforms and development approaches for vaccines in current studies are quite similar, although the immunogenic targets are somehow different. As for animal models, murine strains are the most used models of infection, although there is no consensus model regarding the strains tested, inoculation routes, or challenge dose evaluated. For *B. pseudomallei* vaccination, the inhalational (intranasal or aerosol) and subcutaneous routes are the most common due to the traditional infection routes described in humans, but intraperitoneal has also been used [18]. Surprisingly, for *Shigella*, an enteropathogen, the intranasal inoculation model has been previously used because a similar immunological and pathogenic profile can be established in pulmonary disease that mimics the one observed in human intestinal shigellosis [19]. However, the oral route of inoculation is always more relevant, not only because it is the natural infectious route, but there is no need for medical supplies (e.g., needles), which could be advantageous in developing countries where this pathogen wreaks more havoc. While several of the *Shigella* vaccine studies (Table 1) are advancing to human clinical studies, the *B. pseudomallei* vaccines (Table 2) remain in pre-clinical investigation.

## 2. *Shigella* Vaccines

### 2.1. Inactivated Whole-Cells and Live-Attenuated Vaccines (LAVs)

Whole-cell vaccines offer the advantages of high levels of antigen exposure and the potential to be cross-protective due to the presence of the immunogenic O-antigen and other bacterial surface antigens that are conserved among diverse serotypes [20]. Both inactivated whole-cell and live-attenuated approaches have been developed, with the latter receiving more attention due to better results in efficacy studies.

The inactivated whole-cell approach has been used to develop cross-serotype protective *Shigella* vaccines. One such strain, referred to as Sf2aWC, was developed in *S. flexneri* 2a using the formalin inactivation method [21]. Intranasal (I.n.) vaccination of mice with Sf2aWC resulted in significant levels of serum anti-LPS, anti-IpaB IgG, and anti-LPS IgA. It also conferred protection against further lethal challenge with *S. flexneri* 2a. Additionally, immunization with a trivalent formulation containing Sf2aWC along with formalin-inactivated *S. flexneri* 3a and *S. sonnei* (Sfl3aWC and SsWC, respectively) protected against challenge with all three serotypes, demonstrating the feasibility of a multivalent inactivated whole-cell vaccine [21]. The safety and immunogenicity of Sf2aWC were later evaluated in a phase I study where subjects were orally administered escalating doses of Sf2aWC [22]. All doses were well-tolerated, and the highest dose elicited robust anti-LPS serum IgG and IgA with only transient increases in serum inflammatory cytokines (e.g., IL-17, IFN-γ, TNF-α) and low anti-Ipa antibody levels. Although Sf2aWC offers a safe and promising vaccine to protect against *S. flexneri* infection, a phase II trial was unfortunately withdrawn before participants were enrolled due to lack of funding (clinicaltrials.gov, identifier NCT03038243).

Furthermore, a potential cross-protective inactivated whole-cell vaccine *S. flexneri* 2a strain with truncated LPS was constructed by disruption of the *wzy* gene, which encodes for an O-antigen polymerase, and subjected to formalin inactivation [23]. Intranasal vaccination of mice with a combination of this mutant strain and an adjuvant (double-mutant heat-labile toxin (dmLT) of enterotoxigenic *E. coli*) resulted in significantly higher serum anti-*Shigella* surface protein-1 (PSSP-1), anti-IpaB and anti-IpaC antibodies, and bronchoalveolar lavage fluid IgG antibody compared to immunization with wild-type (WT) formalin-inactivated *S. flexneri* 2a. PSSP-1 is a surface protein that is conserved within the genus, and the truncated LPS of the Δ*wzy* mutant exposes this outer membrane protein, enhancing the antibody response in vaccinated animals [23]. This non-pathogenic formulation was also able to protect mice from lethal challenge with various *Shigella* strains including *S. flexneri* and *S. sonnei*, which confers a cross-serotype protection without the need for a multi-species combination.

The *Shigella* T3SS is an important pathogenic feature associated with the intracellular lifestyle and different live-attenuated vaccines in the development of target genes involved in this system. One of the most promising live-attenuated *Shigella* vaccines is WRSS1, which is a *S. sonnei* strain Moseley that lacks intercellular spreading ability due to the loss of the virulence plasmid-encoded gene *virG* [24]. The protein VirG (also known as IcsA) is essential for the polymerization of actin at one of the bacterial poles and is responsible for intracellular and intercellular movement [25]. This attenuated strain has been used for other vaccines in development, known as WRSs2 and WRSs3, which also harbor additional deletions to increase attenuation and enhance their safety [26]. Both vaccines lack plasmid-encoded enterotoxin ShET2-1 and its paralog ShET2-2 (*senA* and *senB* genes, respectively), which are involved in early fluid secretion, while the bacteria pass through the small intestine. WRSs3 harbors another deletion in the plasmid-encoded *msbB2* gene, which results in less hexacylated lipid A, which is associated with decreased pathology and reactogenicity [27]. Phase I clinical trials demonstrated that lower oral doses of WRSS1 were well tolerated and immunogenic, while higher doses were associated with increased reactogenicity, including diarrhea and fever [28,29]. This was also seen in ∆*virG* mutants derived from *S. flexneri* 2a and *S. dysenteriae* type 1, known as SC602 and WRSd1, respectively [30,31]. WRSs2 and WRSs3 have been also evaluated in a phase I clinical trial using oral administration in doses ranging from 10^3^ to 10^7^ CFU [26]. Both strains were well tolerated and safe in all doses, while moderate to severe diarrhea was observed in three patients receiving the highest dose. Patients mounted significant serum IgG and IgA, as well as fecal IgA titers in a dose-dependent manner. However, the humoral response dropped to baseline titers 8 weeks post-inoculation [26]. Although these vaccines offer a relatively safe immunogenic option, more studies are required to further reduce unwanted side effects and to determine if they retain the same safety profile in children.

Other attenuated *Shigella* vaccine strains have been pre-clinically evaluated for the induction of serotype-independent responses. Deletion of the *hfq* gene, which encodes an RNA-binding protein, in *S. flexneri* 2a results in attenuation due to the repression of stress response regulators, which is associated with a lack of *virB* T3SS regulator [32]. Ocular vaccination in guinea pigs resulted in protection against subsequent ocular challenge with *S. sonnei* and *S. dysenteriae* as well as an oral challenge with *S. sonnei*, showing protective immune responses against various serotypes. Oral vaccination induced significant levels of *S. flexneri* 2a-specific IgG and IgA, with cross-reactive antibodies against several strains of *Shigella* and an enteroinvasive *E. coli* (EIEC) strain, suggesting that vaccination against multiple related enteric pathogens is plausible [32]. In fact, another potential cross-protective live-attenuated strain of *S. flexneri* 2a was created by removing genes involved in LPS O-antigen expression (*rfbF*), invasins (*ipaB* and *ipaC),* and ShET-1 enterotoxin expression (*setBA*) while simultaneously expressing two fused enterotoxigenic *E. coli* (ETEC) antigens: heat-labile enterotoxin subunit B (LT-B) and detoxified heat-stable toxin (ST) [33]. This vaccine strain, ShigETEC, was found to be non-invasive, non-pathogenic, and protected mice from a lethal intranasal challenge with both *S. sonnei* and *S. flexneri* 6.

*Shigella* and ETEC-specific responses were also seen in mice with another combined vaccine constructed using the live-attenuated *Shigella* strain CVD 1208S [34]. This strain is a *S. flexneri* 2a auxotroph derivative with deletions in the *guaBA* operon, as well as *set* and *sen* genes. The *guaAB* operon is required for de novo guanine nucleotide biosynthesis and intracellular survival [35,36]. This strain was well-tolerated in humans up to 10^9^ CFU through oral inoculation, inducing in all the patients an anti-LPS IgA response and mounting an anti-LPS IgG response in 70% of subjects, while more than half of them presented symptoms (headache, abdominal cramps, malaise, etc.) [34,37]. More recently, the same deletions have been introduced in other strains of *S. flexneri*: *S. flexneri* 3a (referred to as CVD 1213) and *S. flexneri* 6 (CVD 1215) [38]. Both strains showed attenuation in the Serény test, a keratoconjunctivitis in the guinea pig model, which is used to demonstrate *Shigella* pathogenicity and test the efficacy of vaccine candidates [39]. They were still able to stimulate cytokine production from epithelial cells and macrophages and induce robust serotype-specific antibody responses following the I.n. immunization of guinea pigs. The immunization of each strain produced homologous protection in those animals, and a mixture of all three strains provided cross-protectiveness against each virulent wild-type strain of *S. flexneri* [38]. This study indicated that these attenuated strains could be combined to create a vaccine capable of protecting against various serotypes of *S. flexneri*. Unfortunately, phase IIa and IIb trials utilizing the CVD 1208S strain were terminated due to its reactogenicity, and further modifications will be needed to increase safety (https://clinicaltrials.gov/, accessed on 1 August 2021, identifier NCT00866476 and NCT00866242). Using that strain as a backbone, the ETEC operon encoding CFA/I, a colonization factor used for adherence to the intestinal human cells, and genes encoding the LTb A2 and B subunits were engineered into the chromosome, creating the strain CVD 1208S-122 [40]. Intranasal immunization with this strain induced both *Shigella* and ETEC-specific IgG serum antibodies in mice and protected them from weight loss following oral infection with either *S. flexneri* or ETEC. A phase I human clinical study evaluating the safety and immunogenicity of CVD 1208S-122 is in progress (https://clinicaltrials.gov/, accessed on 1 August 2021, identifier NCT04634513).

### 2.2. Subunit and Glycoconjugate Vaccines

Natural *Shigella* infection typically elicits a serotype-specific protective immune response toward the O-antigen; however, antibodies specific for other *Shigella* antigens, such as those encoded by the virulence plasmid, have been identified in patients [41]. Subunit-based preparations containing proteins conserved among various *Shigella* serotypes have been used to enhance serotype-independent responses. The most popular targets for these types of vaccines include T3SS proteins evaluated alone or in combination with adjuvants [42,43,44,45,46].

The Invaplex_NAT_ vaccine was produced via the purification of water-extractable antigens from invasive *S. flexneri* 2a and contained the invasion proteins IpaB and IpaC, serotype-specific LPS, and other non-immunogenic proteins [42]. Purified Invaplex_NAT_ was shown to be immunogenic and protective in both mouse and guinea pig models. Phase I clinical studies have shown that it is safe, well-tolerated, and immunogenic in humans [43]. A synthetic Invaplex, termed Invaplex_AR_, was produced with purified LPS and recombinant IpaB and IpaC using molar ratios of the components from purified Invaplex_NAT_ [44]. It contained greater quantities of the three antigens and induced higher serum IgG and IgA antibody responses to IpaB and IpaC proteins in mice and guinea pigs compared to Invaplex_NAT_ and provided better protection in mice. Importantly, the incorporation of the LPS of *S. sonnei* instead of *S. flexneri* into Invaplex_AR_ provided cross-species protection against I.n. challenge with both *S. sonnei* and *S. flexneri* 2a [44].

Individual T3SS proteins have also been pre-clinically tested as vaccine candidates. Intranasal and intragastric immunizations with the invasion protein IpaD, a needle-tip protein of the T3SS, elicited protein-specific serum IgG and IgA responses and protected mice from subsequent I.n. challenge with *S. flexneri* 2a [45]. In the same study, SipD, the needle-tip protein of the *Salmonella* Typhimurium T3SS, an IpaD homolog, provided protection against oral challenge with *S.* Typhimurium and I.n. challenge with *Shigella*, indicating a role of this formulation for a cross-protective vaccine. A fused protein containing recombinant *Shigella* IpaB and *S.* Typhi GroEL was evaluated in a mouse model for immunogenicity and protective efficacy [46]. GroEL is a well-known immunogenic heat shock protein induced during stressful conditions (i.e., macrophage infection) and is used as an adjuvant [47]. Intranasal immunization of mice with IpaB-GroEL stimulated higher serum and mucosal antibody responses compared to the co-administration of each recombinant protein and protected against subsequent lethal challenge with *S. flexneri* 2a, *S. sonnei*, and *S. boydii* [46].

Chromosomally-encoded proteins have also been found to be immunogenic, including three autotransporters that are encoded on the pathogenicity island SHI-1: SigA, Pic, and Sap [48]. SigA and Pic are serine-protease autotransporters involved in virulence and immune recognition [49]. The Sap protein is still uncharacterized but has high sequence similarity to the gene encoding antigen 43, an autotransporter involved in *E. coli* autoaggregation [50]. A multivalent vaccine, called rMESF, containing a chimeric protein derived from the immunodominant epitopes from SigA, Pic, and Sap bound to GroEL of *S*. Typhi as an adjuvant was used to I.n. immunize mice [48]. This vaccine elicited robust, rMESF-specific serum IgG and IgA and fecal IgA titers, and splenocytes from immunized mice elicited significant levels of TNFα, IL-17, and IFN-γ. Lastly, immunization with rMESF provided 100% protection in mice against lethal I.n. challenge of *S. flexneri* [48].

Outer membrane proteins (OMPs) of *Shigella* species have been evaluated in multiple studies as subunit vaccines. OmpA is conserved and cross-reactive with multiple strains of *S. flexneri* and I.n. immunization in mice elicited OmpA-specific serum IgG and IgA [51]. It also protected mice from lethal I.n. challenge with *S. flexneri* 2a [52]. The vaccine candidate EpiMix was produced by combining five synthetic epitopes derived from OmpA and OmpF conjugated to ovalbumin [53]. Intramuscular immunization of mice with EpiMix induced specific serum IgG and fecal IgA and protected mice from developing shigellosis following intraperitoneal (I.p.) challenge with *S. flexneri* 2b. Additionally, splenocytes taken from immunized mice produced significant levels of IFN-γ when stimulated with EpiMix, compared with non-immunized controls. Synthetic epitopes of the OMP OmpC were also evaluated for immunogenicity [54]. Antibody responses to the synthetic linear or cyclic peptides of the main OmpC epitope, conjugated with the tetanus toxoid (TT) as adjuvant, were compared, and there was better recognition of OmpC from antibodies against the cyclic-TT peptides [54]. However, in vivo studies need to be conducted to determine if these synthetic OmpC peptides might be protective.

In general, conjugate vaccines consist of capsular polysaccharides chemically conjugated to a protein carrier. However, conjugate vaccines for *Shigella* utilize the LPS O-antigen because during natural infections, it elicits serotype-specific, short-lived protective antibodies [55,56]. One conjugation method that has been used in several studies is in vivo conjugation of *Shigella* O-antigen to *Pseudomonas aeruginosa* exotoxin A (EPA) using an *E. coli* glycosylation method [57,58]. This involves functionally expressing the N-linked glycosylation system from *Campylobacter jejuni* in *E. coli* along with the carrier protein EPA. When the expression of the *Shigella* O-antigen is also incorporated, it is enzymatically conjugated, generating the O-antigen-EPA complex that can be extracted and purified from the *E. coli* cells [57,58]. Many *Shigella* vaccine studies utilizing this platform have been completed, including a phase III trial using *S. sonnei* O-antigen-EPA [59,60,61,62]. This vaccine was found to be safe and immunogenic in both adults and children, while protection was only significant in children older than 3 years of age.

Clinical trials using the same carrier protein and conjugation technology have also been performed for *S. flexneri* and *S. dysenteriae.* The Flexyn2a (O-antigen from *S. flexneri* 2a) was evaluated for safety and immunogenicity in a phase I study, where subjects received two intramuscular (I.m.) injections of Flexyn2a with or without alhydrogel as adjuvant [60]. The vaccine was well-tolerated and only associated with mild adverse events. It elicited significant LPS-specific serum IgG and IgA, regardless of the presence of the adjuvant, and sera from immunized individuals were demonstrated to have bactericidal activity against *S. flexneri* 2a. Flexyn2a was further evaluated in a human challenge study where participants received two doses of the vaccine followed by oral challenge [61]. Although vaccination only resulted in around a 30% reduction in shigellosis compared to subjects receiving a placebo, the efficacy was higher in protecting against severe disease (around 51%). The protection was associated with LPS-specific IgG responses. In vaccinated subjects that developed the disease, the severity was lower, and they were less likely to need antibiotic intervention. Lastly, the bioconjugate vaccine strain GVXN SD133 was created in the same manner and contained the O-antigen from *S. dysenteriae* type 1 [62]. A phase I study using I.m. administration of GVXN SD133 determined that it was well-tolerated with safety and reactogenicity profiles similar to those of other conjugated vaccines.

While O-antigen-EPA bioconjugates have been proven as potential *Shigella* vaccine candidates capable of stimulating serotype-specific responses, other conjugation methods have also been used for pre-clinical and clinical studies. The most recent study was a phase I trial employing a synthetic carbohydrate-based conjugate vaccine, called SF2a-TT15 [63]. The carbohydrate component, a 15-mer oligosaccharide identified from a synthetic O-antigen library as the best antigenic, structural, and conformational mimic of *S. flexneri* 2a O-antigen, was conjugated to tetanus toxoid. After 3 I.m. injections, the vaccine was found to be well-tolerated, with no severe adverse events reported. It also induced significant anti-*S. flexneri* 2a LPS IgG titers compared to placebo, and sera from vaccinated subjects had bactericidal functionality. A phase IIa clinical study evaluating safety and immunogenicity in both adults and children is in progress (https://clinicaltrials.gov/, accessed on 1 August 2021, identifier NCT04056117). A non-toxic mutant of diphtheria toxin, called cross-reactive material (CRM_197_), has also been used for conjugation to *S. flexneri* 2a O-antigen [64]. This glycoconjugate was discovered to be non-toxic during in vitro assays and had an extended shelf-life, but the immunogenicity of this formulation has yet to be evaluated. Furthermore, a potential trivalent vaccine for *S. flexneri* 2a, *Campylobacter jejuni*, and ETEC was constructed by administering the following combination formulation: detoxified *S. flexneri* 2a O-antigen conjugated to the CFA/I fimbriae proteins from ETEC, HS23/36 capsular polysaccharide from *C. jejuni* conjugated to the CFA/I fimbriae proteins from ETEC, and HS3 capsular polysaccharide conjugated to colonization factor proteins encoded by the CS6 operon from ETEC. The vaccine was immunogenic and elicited IgG responses to all included antigens in mice when administered subcutaneously (S.c.), but protective efficacy was not assessed [65].

### 2.3. Outer Membrane Vesicles (OMVs)

Several studies have proven that OMVs of *Shigella* are both immunogenic and protective [66,67,68]. One study assessed the physiochemical characteristics, protein content, toxicity, biodistribution, and protectiveness of heat-induced OMVs (HT OMVs) of *S. flexneri* 2a compared to naturally produced OMVs [69]. These are generated due to heat-induced changes between the outer and inner membrane layers, triggering the release of these vesicles. Their composition regarding the cytosolic components is similar to the naturally generated OMVs, supporting the idea that they are not the result of a cells lysis, while their OMPs composition is enhanced (OmpA, OmpX, and OmpW) [69]. These HT OMVs showed that they were more bioavailable in the gut after I.n. administration in the in vivo model and provided better protection to mice following I.n. challenge with the homologous *S. flexneri* strain [69]. In addition, OMVs from *Salmonella* were engineered to express *S. flexneri* 2a O-antigen and utilized as a vaccine vector [70]. Intranasal and I.p. immunization in mice using this vaccine induced significant anti-*Shigella* LPS serum antibodies and provided protection against virulent *S. flexneri* challenge.

Another strategy for OMV vaccine development is the production of generalized modules for membrane antigens (GMMAs), which involves genetically enhancing bacteria to increase particle production and thus the immunogenic components LPS and OMPs [71]. For *Shigella*, this involves the deletion of the *tolR* gene, which is implicated in linking the inner and outer membranes [72]. A *S. sonnei* GMMA-based vaccine, called 1790GAHB, was genetically modified to produce penta-acylated LPS with decreased endotoxicity due to deletion of the late acyltransferase gene *htrB*, along with deletions in *tolR* and *virG* [73]. Purified GMMAs were formulated with alhydrogel as an adjuvant and demonstrated to be immunogenic in mice when given I.p. and rabbits through different routes. Immunization elicited anti-LPS IgG antibodies, with no observed local or systemic toxicology in rabbits. This vaccine strain progressed to phase I clinical trials in which subjects were given escalating doses of the vaccine via I.m., intradermal (i.d.), and I.n. [74]. Intramuscularly administered vaccine was well-tolerated and stimulated antibodies to *S. sonnei* O-antigen. Vaccine administered I.d. or I.n., although well-tolerated, were poorly immunogenic at the delivered doses. GMMAs derived from *S. flexneri* 6 were compared to the *S. flexneri* 6 O-antigen conjugated to CRM_197_ [75]. In mice, subcutaneous (S.c.) immunization of *S. flexneri* 6 derived GMMAs combined with alhydrogel elicited similar levels of persistent anti-O-antigen IgG with bactericidal activity as compared to mice immunized with the glycoconjugate, but the GMMAs elicited higher antibody responses when they were not combined with the adjuvant.

### 2.4. Reverse Vaccinology

Reverse vaccinology is an in silico approach that involves bio- and immunoinformatics to select potential bacterial antigens based on qualities such as cellular location, predicted T and B cell epitopes, and conservation among strains/serotypes [76]. This method has been used to identify novel *Shigella* protein antigens that can induce serotype-independent protection. One such antigen, FimG, a type 1 fimbrial protein, was discovered and became a new efficacious vaccine candidate [77]. Scanning of the *S. flexneri* 2a proteome in this study revealed seven outer membrane or extracellular proteins that were conserved among various *Shigella* serotypes but did not have homology with human proteins. FimG was selected as the best candidate due to its high epitope scores and its potential to be the most immunogenic. Mice I.p. immunized with recombinant FimG elicited robust antigen and whole-cell specific IgG responses, and they were protected from subsequent I.p. challenge with *S. flexneri* 2a.

Another research group analyzed *S. flexneri* 2a OMPs and identified five proteins (FepA, OmpC, TolC, NlpD_1, and NlpD_2) with high antigenicity scores that are conserved among other *Shigella* serotypes and do not share homology with human proteins [78]. They also predicted the B and T cell epitopes of each protein but did not demonstrate immunogenicity in mice. FepA, an outer membrane siderophore receptor, was also identified in another study utilizing a different reverse vaccinology approach [79]. Predicted immunogenic *Shigella* antigens were expressed and printed on a microarray and then probed with sera from patients with confirmed acute or convalescent infections. FepA was observed to have high IgG sero-reactivity with all sera as well as from sera from infants born to mothers with high *Shigella* titers. Although the protective efficacy of FepA from *Shigella* has not been demonstrated in vivo, mice I.p. immunized with pathogenic *E. coli* FepA, which has 98% homology with *S. flexneri* FepA, did produce antigen-specific serum antibody titers and were protected from lethal I.p. challenge with both *E. coli* and *S. flexneri* [80].

The proteome of *S. sonnei* has been evaluated for potential vaccine candidates as well. Three essential *S. sonnei* OMPs, known as TolC, PhoE, and outer membrane porin protein, were identified [81]. Additionally, the predicted B and T cell epitopes of each protein were found to be conserved in four completely sequenced strains of *S. sonnei*, and the structural analysis of each epitope revealed deep binding to the binding groove of human allele HLA-DRB1*0101. Future in vivo studies are warranted to determine the usefulness of these proteins as subunit vaccines.

### 2.5. Others (Non-Pathogenic Bacteria as Vectors)

Another approach used to deliver *Shigella* antigens is the *Salmonella enterica* serovar Typhi Ty21a vector [82]. Ty21a is a live-attenuated vaccine strain that is currently licensed to protect against diarrhea caused by *S. Typhi* [83]. Initial clinical trials using a Ty21a expressing the *S. sonnei* O-antigen on a plasmid proved to be safe and immunogenic; however, the plasmid was unstable and demonstrated lot-to-lot variation [84,85]. To mitigate this, a recent study incorporated the form 1 O-antigen gene cluster from *S. sonnei* into the chromosome of Ty21a and co-expressed with *Shigella* glutaminase-glutamate decarboxylase systems to create a more acid-resistant strain [86]. Mice I.n. immunized with this strain induced both *Shigella* and *Salmonella* antibodies and survived lethal I.n. challenge with *S. sonnei*. A similar study was completed but instead incorporated *S. dysenteriae* type 1 O-antigen biosynthetic genes from two separate genetic loci, *rfp* and *rfb*, into the chromosome of Ty21a [87]. Intraperitoneal administration of this strain in mice elicited serum IgG against both *S. dysenteriae* and *S.* Typhi LPS and protected from subsequent lethal I.p. challenge with *S. dysenteriae.* Finally, I.p. immunization of mice with Ty21a engineered to stably express O-antigen from either *S. flexneri* 2a or 3a elicited anti-LPS antibody titers against each specific serotype LPS, but no cross-reactivity was observed between serotypes 2a and 3a [82]. The mice immunized with the Ty21a vaccine strain expressing either *S. flexneri* 2a or 3a O-antigen showed significant protection against lethal I.p. challenge with virulent *S. flexneri* 2a or 3a, respectively.

The delivery of *Shigella* antigens has also been accomplished via the non-pathogenic, non-colonizing bacteria *Lactococcus lactis.* This system has been used to express OmpA from *S. dysenteriae* type 1, which was then used to orally and intranasally immunize mice [88]. Both routes induced anti-OmpA serum IgG and fecal IgA, with higher levels after oral immunization. Protective efficacy was further evaluated using this vaccine strain, termed LacVax, where oral vaccination protected mice from developing shigellosis following I.p. challenge with *S. flexneri* 2a [89]. Alternatively, *L. lactis* was also used to express a synthesized protein chimera of intimin from enterohemorrhagic *E. coli* (EHEC) and *Shigella* IpaB [90]. Oral immunization with this strain elicited significant titers of chimera-specific serum IgG and IgA and fecal IgA, reduced shedding of EHEC following oral infection, and protected mice from lethal I.n. infection with *S. flexneri.*

## 3. *Burkholderia pseudomallei* Vaccines

### 3.1. Inactivated Whole-Cells and Live Attenuated Vaccines (LAVs)

Heat-killed preparations of different *Burkholderia* whole cells have been used to immunize BALB/c mice, and the various outcomes of protection levels have been reported [91,92,93]. Intraperitoneal immunization of heat-killed *B. pseudomallei* (*Bpm*) strain K96243 and 576 showed 80–100% protection at day 21 against I.p. challenge of both live bacterial strains [93]. The non-pathogenic soil saprophyte, *Burkholderia thailandensis* (*Bt*), and a host-restricted pathogen causing glanders mainly in equines, *Burkholderia mallei* (*Bm*), are closely related species to *Bpm*, and the genome is highly conserved in these three species [94]. Heat-killed whole cells of *Bm* and *Bt* provided 70% and 60% cross-protection, respectively, against strain K96243 when used I.n. route for both vaccination and challenge [93]. However, the protective efficacy appeared to decrease when using inconsistent routes between wild-type challenge and heat-killed *Bpm* immunization, e.g., I.p. immunization of heat-killed *Bm* or *Bt* significantly reduced survival time of mice after *Bpm* aerosol challenge [93]. All heat-killed cell vaccinations generated high IgG antibody titers in BALB/c mice [93]. Moreover, intramuscular vaccination of heat-killed *Bpm* strain A2 failed to protect mice against I.p. challenge [91]. To enhance the protective properties, heat-killed *Bpm* was combined with liposome-nucleic adjuvant, and the result showed 100% protection at day 40 post-challenge [92]. Paraformaldehyde killing was used to prepare *Bpm* vaccine, and I.m. vaccination using this inactivation method showed 50–60% protection at day 30 post-challenge with *Bpm* strain A2 [91].

Live attenuated vaccines (LAVs) are considered the gold standard for melioidosis vaccine research [95]. Single and double mutation strategies of genes encoding crucial proteins in biosynthesis, transport pathways, pathogenesis, and secretion systems have been used for creating attenuated vaccine strains [96,97,98,99,100,101,102,103,104,105]. A single gene mutation of purine biosynthesis (Δ*purN* and Δ*purM*) from transposon interruption in *Bpm* strain E8 showed that Δ*purN* provided better protection against I.p. challenge than Δ*purM* [96]. However, Δ*purN* cannot protect mice from intravenous challenge [96]. In contrast, the deletion of the *purM* gene in *Bpm* strain 1026b (Bp82) showed the potential to confer 60% and 100% protection in BALB/c and C57BL/6 mice, respectively [97]. This study also suggested that humoral immune responses played a critical role in protection, whereas T cells showed a less important role in protection [97]. Another attenuated auxotroph tested was in a subunit of the imidazole glycerol-phosphate (IGP) synthase, which was constructed by deleting a 65 bp of *hisF* gene in *Bpm* MSHR668 [98]. The 668 Δ*hisF* showed highly attenuated phenotype in immunocompromised NOD/SCID mouse strain and protected BALB/c mice during the acute (100% survival) and chronic phase (50%) infection. The high expression of IFN-γ in the vaccination group correlated with protection but not antibody responses [98]. Vaccination with a strain carrying two auxotrophic genes in aromatic compound biosynthesis, Δ*aroB* and Δ*aroC*, was unable to protect BALB/c mice from WT challenge, but only C57BL/6 mice receiving Δ*aroC* showed 20–80% survival for up to 5 months [99,100]. A transposon interrupting the *ilvI* gene (Δ*ilvI*), encoding the subunit of acetolactate synthase enzyme in *Bpm* 2D2, exhibited an attenuated phenotype that provided 80% and 100% protection against *Bpm* strain 576 and BRI challenge, respectively [101]. Furthermore, a T cell depletion study indicated that *Bpm* 2D2 generated CD4^+^ T cells-mediated protective immunity in mice [102]. An auxotrophic of exogenous diaminopimelate (DAP) in *Bpm* 1026b (Δ*asd*) was unable to replicate in HeLa or RAW264.7 cells, and vaccination with this avirulent strain protected BALB/c mice against acute melioidosis, but it failed to protect against chronic melioidosis infection [103].

Due to the intracellular lifestyle and pathogenicity of *Bpm*, important virulent factors have been targeted for countermeasures against this pathogen. A mutation of autotransporter, *batA* gene (Δ*batA*) of *B. mallei* ATCC 23344, conferred 71–100% cross-protection against acute and 67–85% against chronic melioidosis when using *Bpm* 1026b and K96243 as challenge strains [104]. Mutagenesis of the T3SS gene, *bipD*, produced a strain with an attenuated phenotype in BALB/c mice and conferred partial protection (60% survival rate at day 75) against WT challenge [105].

Since most of the single gene mutations of LAVs provided incomplete protection against melioidosis disease, the persistence of vaccine strains in the host is a concern and it also provides a high chance for reversion to WT. These should be critical concerns during development of the next generation of vaccines. Therefore, double gene mutation strategies were used to create *Bpm* LAVs strains [106,107,108,109]. The double deletion mutant of genes encoding (p)ppGpp-synthesis enzyme (Δ*relA* Δ*spoT*) in *Bpm* K96243 provided significant protection for immunized C57BL/6 mice of 100% up to 30 days post-challenge, and the 60% survivor mice remained until day 55, but sterile immunity was not accomplished [106]. The *Bm* and *Bpm* Δ*tonB* Δ*hcp1* double deletion mutant strains were constructed by deleting genes encoding a protein involved in the uptake of iron, *tonB*, and a gene encoding for a protein that is a component of type 6 secretory system cluster 1 (T6SS-1), *hcp1* [107,110]. *Bpm* Δ*tonB* Δ*hcp1* was revealed as the safest and the most efficient LAV strain developed to date because it provides comprehensive evidence of immune responses correlated to protection [107,109]. Intranasal vaccination with *Bpm* Δ*tonB* Δ*hcp1* conferred 100% protection against aerosolized *Bpm* infection in the C57BL/6 mice model of melioidosis, and bacterial clearance in lungs and other target organs was indicative of sterilizing immunity [107]. A recent study illustrated the protective capacity of this vaccine, which generated *Bpm*-specific serum IgM, IgG, and lung IgA and developed diverse polyfunctional memory T cell pools as well as Th1 and Th17 CD4^+^ T cell responses in the lungs and spleens of vaccinated mice [109].

### 3.2. Subunit and Glycoconjugate Vaccines

Subunit vaccines are developed to include only the components or antigens that provide immune stimulation properties without using the entire pathogen. This type of vaccine abolishes the reversion and safety concern of LAVs. Several protein antigens have been identified and evaluated as subunit vaccine candidates against *Bpm* infection [111,112,113,114,115,116]. The ABC transporter proteins LolC, PotF, and OppA were selected and combined with adjuvants and tested against *Bpm* K96243 infection [111]. Intraperitoneal administration of either LolC or PotF, combined with the MPL+TDM adjuvant, protected BALB/c mice 83% and 50%, respectively at day 42 post I.p. challenge. In addition, subcutaneous vaccination of the CpG adjuvant together with LolC afforded better protection when a larger challenge dose was used. Strong antibody and cell-mediated immune responses were stimulated when the protein LolC was combined with complex adjuvants such as MPL-TDM or ISCOM-CpG ODN [111].

Outer membrane proteins are involved in virulence and immunogenicity, resulting in potential targets as subunit vaccine candidates [112,113,114]. When two OmpAs, including Omp3 and Omp7 proteins, were purified, they were recognized by sera from melioidosis patients, and both proteins induced IgG and IgG subclasses, as well as IgM antibody responses upon vaccination; however, they only conferred 50% protection at day 21 post-challenge with *Bpm* strain D286 [112]. Recombinant Omp85 induced a Th2-bias immune response in immunized BALB/c mice, and anti-rOmp85 antibodies were able to promote complement-mediated killing and enhance the opsonophagocytic activity of *Bpm* by human polymorphonuclear cells (PBMCs) [113]. All these immunogenic properties of Omp85 supported its ability to protect up to 70% of immunized mice and reduce bacterial loads in blood and other target organs [113]. The homologous *Bpm* OmpW protein given together with the Sigma Adjuvant system (SAS) triggered Th1-immune response and conferred 75% protection in BALB/c (at day 21) and C57BL/6 (at day 80) mice [114].

Proteins involved in pathogenesis and virulence factors such as the T6SS (T6SS-1) protein Hcp, the T3SS protein BopA, and the autotransporter protein BimA have been examined and evaluated for their potential to serve as melioidosis-specific subunit vaccine candidates [115,116]. As subunit vaccines, each individual recombinant Hcp protein (Hcp1, Hcp2, Hcp3, Hcp4, and Hcp6) was mixed with SAS adjuvant, and BALB/c mice were immunized and subsequently challenged with *Bpm* K96243 [115]. The results indicated that the Hcp proteins failed to protect mice from lethal dose infection as well as their inability to prevent chronic colonization [115]. The mixed adjuvant ISCOM+CpG together with recombinant BopA or BimA proteins from *B. mallei* were investigated to immunize BALB/c mice against melioidosis infection. Immunization with BopA protein was able to induce 60% cross-protective activity, while BimA protein showed only 20% (at day 50 post-infection) [116].

Polysaccharide-based glycoconjugate vaccines have been developed to minimize safety issues and to stimulate both protective antibody and T cell responses [117]. Capsular polysaccharide (CPS) and lipopolysaccharide (LPS) are virulence factors for pathogenic *B. mallei* and *Bpm* and common cell surface polysaccharides used to conjugate with other protective antigens against melioidosis [118,119,120,121]. Immunization with a mixture of CPS and LolC protein conferred significant protection with 70% of mice surviving until day 35 post-challenge; however, this combination could not reduce bacterial load in organs of immunized mice [122]. A combination of covalently linked conjugated CPS + CRM197 (recombinant diphtheria toxin mutant) plus Hcp1 or TssM protein offered robust protective 100% efficacy (with 70% sterilizing immunity) and 80%, respectively, against aerosol melioidosis in C57BL/6 mice [120]. The immune response analysis data have shown that the CPS-CRM197 induce IgG that has the potential to be an opsonizing antibody, while Hcp1 and TssM induced both IgG antibody and IFN-γ secreting T cell responses [120]. Carrier protein, non-toxic Hc domain of tetanus toxin (TetHc) has been also used to couple with a chemically synthesized hexasaccharide fragment of *Bpm* CPS (TetHc-_SH_CPS) [121]. BALB/c immunization with TetHc-_SH_CPS via the I.p. route showed 66.7% survival at day 35 after I.p. challenge with *Bpm* K96243 and immunized sera contained IgM and IgG antibodies, which can recognize purified, native CPS [121]. *Bpm* LPS conjugated to TetHc (TetHc-LPS) elicited antigen-specific IgG toward Th1 immune responses, and 81% survival with significant reduction in bacterial load was found in TetHc-LPS immunized mice [119]. In addition, *Bpm* O-polysaccharide (OPS) with carrier protein AcrA generated an IgG immune response, but only partial protection was observed with 40% survival on day 12 post-infection [118].

### 3.3. Outer Membrane Vesicles (OMVs)

Multivalent OMVs derived from *Bpm* 1026b have been evaluated for their efficacy to confer protection in a murine model of melioidosis [123,124,125]. *Bpm* OMVs with a size range between 50 and 250 nm in diameter were purified from strain 1026b after growing in liquid nutrient-rich LB. In this culture condition, the OMVs produced contained several protein antigens, including periplasmic, outer membrane, and extracellular proteins [123]. Furthermore, numerous immunogenic proteins were recognized by convalescent sera from rhesus macaques infected by *Bpm* [123]. Subcutaneous immunization of the purified OMVs vaccine into BALB/c provided significant protection against aerosol and I.p. challenge with *Bpm* strain 1026b and heterologous strain K96243, respectively [123,124]. *Bpm* OMVs vaccine induced OMV-, LPS-, and CPS-specific IgG, IgM, and IgA antibody responses as well as type 2 antibody responses [123,124]. In addition, OMVs immune serum was able to promote the killing of *Bpm* in vitro, and it was sufficient to confer protection against *Bpm* challenge in vivo [124]. In order to advance the OMVs vaccine, the safety and immunogenicity were further evaluated in rhesus macaques by using a prime and two boosts vaccination protocol with escalating doses of OMVs together with CpG oligodeoxynucleotide (ODN) adjuvant [126]. Blood chemistry and clinical measurements of immunized rhesus macaques indicated that the vaccine was safe at the site of injection and did not cause dysfunction of liver or kidney. Analysis of plasma collected from immunized macaques revealed significant increases of *Bpm* OMV-, LPS-, and CPS-specific IgG antibodies, which suggested that the vaccine can confer protection against infection [126]. A recent study demonstrated that *Bpm* OMVs grown in macrophage-mimicking intracellular environment conditions (M9 minimal media depleted from zinc and iron) produced different and more diverse intracellular-bound virulence proteins compared to LB media [125]. Particularly, the presence of highly immunogenic and protective antigens, Hcp-1 and other T6SS-1 and T3SS-3 effector proteins, were observed in M9 OMVs but not LB. Compared to the *Bpm* Bp82 LAV, M9 OMVs showed similar protective efficacy in C57BL/6 mice after challenge with aerosolized *Bpm*. M9 OMVs have been shown to induce both humoral (specific-IgG) and cellular (IFN-γ and IL-17 producing CD4^+^, and IFN-γ producing CD8^+^ T cells) immune responses. Additionally, OMVs were taken up by dendritic cells (DC) in vivo and consequently drive DC activation and maturation [125].

### 3.4. Reverse Vaccinology and Nanovaccines

Reverse vaccinology approaches have been used to identify new immunogenic antigens and evaluate them as potential vaccine targets against melioidosis disease [127,128,129,130,131,132]. The vaccine candidates were selected based on protein subcellular localization, topology, antigenicity, epitopes, and its binding to the major histocompatibility complex (MHC) class I and II molecules [130].

Combined subtractive genomics and reverse-vaccinology strategies have been used to identify antigenic peptide sequences from the secretory pathway protein SecF of *Bpm* strain Bp1651. The SecF protein was predicted to be a potential vaccine candidate that interacted with the human HLA receptor [128]. A combination of epitope design by computational and in vitro immunological experiments demonstrated the presence of a highly immunologic epitope 3 of BPSL2765, which is an acute phase antigen. An epitope 3 was recognized by serum from recovering melioidosis patients, and anti-epitope 3 antibody specifically agglutinated *Bpm* [131]. A similar strategy was used to identify and confirm the ability of type I fimbrial subunit, BPSL1626 antigen, to induce T cell responses, and it was recognized by serum antibodies from melioidosis patients [132].

The reverse vaccinology approaches together with multicomponent nanovaccines have been recently used to advance vaccine development against *Bpm* infections in animal models [127,129]. Protein candidates, including Hemagglutinin, Hcp1, and FlgL were predicted by using a combination of bio- and immunoinformatics approaches, and they showed seropositive responses with melioidosis sera from human and animal origin [127]. Individual or combination (combo) proteins were conjugated with gold nanoparticles (AuNP) along with the LPS from *B. thailandensis*. Immunization of C57BL/6 mice with AuNP-FlgL-LPS and AuNP-combo-LPS glycoconjugates provided 90–100% protection at day 35 post-challenge with *Bpm* K96243. A significant reduction of bacterial burden in organs and high protein- and LPS-specific IgG were observed in immunized mice [127]. Exploiting the use of an AuNP-based glycoconjugate platform to generate protective vaccines against *Bpm* was further studied with additional predicted immunogenic proteins, including OmpW and the porins (OpcP and OpcP1) [129]. Intranasal immunization of C57BL/6 with individual porin proteins coupled with LPS (Au-OpcP-LPS or Au-OpcP1-LPS) and CpG adjuvant provided the highest protection against *Bpm* infection (up to 90% at day 35 post-infection); however, the combination of these proteins demonstrated the enhancing protective properties by affording 100% protection. The humoral immune response analysis demonstrated that serum from Au-OpcP-LPS or Au-OpcP1-LPS immunized mice induced strong antigen-specific IgG (mainly IgG2c), which promoted opsonophagocytic activity by primary murine macrophages. In addition, the protein combination also elicited antigen-specific IgG and IgA in lung as well as mixed Th1–Th17 cytokine responses after restimulation with antigens [129].

### 3.5. Others (DNA Vaccines and Viral Vector-Based Vaccines)

Plasmid DNA has been used to develop new vaccine candidates. The plasmid DNA encoding flagellin protein was modified by the addition of two CpG motifs (immunostimulatory) [133]. The plasmid carrying *fliC* DNA only (pcDNA3/*fliC*) and in combination with CpG (pcDNA3/CpG-*fliC*) were compared in the context of protection and immune responses in BALB/c mice. Immunization with CpG-modified DNA provided higher percent protection and significantly lower bacterial load in spleen and liver compared to pcDNA3/*fliC* immunized mice after being challenged with a mixture of 16 *Bpm* strains. This restricted bacterial growth in immunized mice was a consequence of the induction of Th1 immune response (IgG2a and IFN-γ secretion) to CpG and flagellin stimulation. Furthermore, *Bpm* flagellin DNA vaccines were used by employing pVAX1 vector with either codon optimization for translation efficiency and ribosomal binding (Kozak), cellular secretion signal (hTPA), or endoplasmic reticulum signal (KDEL) [134]. Three doses of the rapid tattoo vaccination of pVAX-hTPA-FliC provided the most effective DNA vaccine that induced anti-FliC IgG levels in plasma and reduced bacterial loads in the blood, lung, liver, and spleen of C57BL/6 mice. However, a single I.n. vaccination with pVAX-hTPA-FliC was more effective than a single tattoo vaccination in lowering bacterial loads and decreasing pulmonary cytokine levels, lung pathologic scores, systemic inflammation, and organ damage after I.n. challenge with *Bpm*. Nevertheless, I.n. vaccination with pVAX-hTPA-FliC was suggested to be equally effective as the S.c. route of the recombinant FliC protein, but it showed only partial protection as well as undetectable FliC-specific IgG responses [134].

A viral vector-based vaccine has been also developed against *B. mallei* and *Bpm* infections using the platform of Parainfluenza Virus 5 (PIV5) to deliver the highly conserved *Burkholderia* surface immunogenic antigen, autotransporter protein BatA [135]. A single-dose vaccination of recombinant PIV5 expressing BatA protein was able to protect BALB/c mice, 80% and 60%, from acute and chronic infection of *Bpm*, respectively. The analysis of serum and stimulated splenocytes collected from vaccinated animals suggested that the T cell responses played an important role in the protection and elimination of *Bpm* from lungs and spleens, whereas absent to low antibody titers against BatA indicated a minor role of humoral immune responses.

## 4. Conclusions

The development of vaccines against intracellular bacterial pathogens is challenging due to the incomplete understanding of the mechanism that grants effective immune responses. In this review, we compare the current approaches used in vaccine development against *Shigella* and *B. pseudomallei*. Even though these two intracellular pathogens cause different disease outcomes, the vaccine platforms are evaluated based on protective and immunogenic antigens inducing both humoral and cellular immune responses. *Shigella* vaccine studies have demonstrated more effectiveness and have advanced to human testing, while *B. pseudomallei* vaccines have mostly been tested in pre-clinical trials with animal models. The protection against chronic and persistent forms of melioidosis disease is a challenge for further vaccine development. Ongoing studies will provide more significant information that can lead to licensed safe and efficacious *Shigella* and *B. pseudomallei* vaccines to prevent infection as well as reduce the mortality rate worldwide, especially in developing countries.

## Figures and Tables

**Table 1:** List of *Shigella* vaccines.

Vaccine Type	Antigens/Mutant	Immunization Route	Pre/Clinical	Status	Ref.
**Inactivated**	*S. flexneri* 2a (formalin)	Oral	Phase IIa/IIb (Sf2aWC)	W	[21,22]
	*S. flexneri* 2a ∆*wzy* (formalin)	I.n.	Preclinical	-	[23]
**LAVs**	*S. sonnei* Moseley ∆*virG*	Oral	Phase I (WRSS1)	C	[24,28,29]
	*S. sonnei* Moseley ∆*senA* ∆*senB* ∆*virG*	Oral	Phase II (WRSs2)	ID	[26]
	*S. sonnei* Moseley ∆*senA* ∆*senB* ∆*virG* ∆*msbB2*	Oral	Phase I (WRSs3)	C	[26]
	*S. flexneri* 2a ∆*virG* ∆*iuc*	Oral	Phase I (SC602)	-	[30]
	*S. dysenteriae* type 1 ∆*virG* ∆*stxA* ∆*stxB*	Oral	Phase I (WRSd1)	-	[31]
	*S. flexneri* 2a ∆*guaBA* ∆*sen* ∆*seT*	Oral	Phase IIa/IIb (CVD 1208S)	T	[34,37]
	*S. flexneri* 3a ∆*guaBA* ∆*sen*	-	Preclinical (CVD 1213)	-	[38,39]
	*S. flexneri* 6 ∆*guaBA*	-	Preclinical (CVD 1215)	-	[38,39]
	*S. flexneri* 2a ∆*hfq*	Oral	Preclinical	-	[32]
	*S. flexneri* 2a ∆*rfbF* ∆*setBA* ∆*infA* ∆*ipaBC*::*infA*-3×[LTB-ST_N12S_]	I.n.	Preclinical (ShigETEC)	-	[33]
	*S. flexneri* 2a ∆*guaBA* ∆*sen* ∆*seT*::CFA/I-LT_A2_-LT_B_	I.n.	Phase I (CVD 1208S-122)	ID	[40]
**Subunit**	*Shigella* LPS-IpaB-IpaC	I.m.	Phase I (Invaplex_NAT_)	C	[43]
	*Shigella* LPS-IpaB-IpaC	I.m.	Phase I (Invaplex_AR_)	C	[44]
	*Shigella* IpaB-GroEL	I.n.	Preclinical	-	[46]
	*S. flexneri* 2a OmpA	I.p.	Preclinical	-	[52]
	*Shigella* OmpA-OmpC-OVA	I.m.	Preclinical (EpiMix)	-	[53]
	*Shigella* SigA-Pic-Sap	I.n	Preclinical (rMESF)	-	[48]
**Glycoconjugate**	*S. sonnei* and *S. flexneri* 2a O-SP-rEPA	I.m.	Phase III	C	[60]
	*S. flexneri* 2a O-EPA	I.m.	Phase IIb (Flexn2a)	C	[61,62]
	*S. dysenteriae* O1-EPA	I.m.	Phase I (GVXN SD133)	C	[63]
	*S. flexneri* 2a O-SP	I.m.	Phase IIa (SF2a-TT15)	IP	[64]
	*S. flexneri*-LPS-CFA/I-HS23/36	S.c.	-	-	[65]
	*S. flexneri* 2a OPS-CRM_197_	-	-	-	[66]
**OMVs**	*S. flexneri* HT-OMV	I.n.	Preclinical	-	[70]
	*Salmonella* OMV-*S. flexneri* 2a O-antigen	I.n./I.p.	Preclinical	-	[71]
	*S. sonnei* NCGH1790 GMMA	I.n.	Preclinical (1790GAHB)	-	[74,75]
	*S. flexneri* 6 GMMA-CRM_197_	S.c.	Preclinical	-	[76]
**Reverse vaccinology**	*S. flexneri* 2a FimG	I.p.	Preclinical	-	[78]
	*Shigella* FepA	I.p.	Preclinical	-	[81]
**Others**	*S.* Typhi Ty21a vector *S. sonnei* O-antigen	I.n.	Preclinical	-	[87]
	*S.* Typhi Ty21a vector *S. dysenteriae* type 1 O-antigen	I.p.	Preclinical	-	[88]
	*S.* Typhi Ty21a vector *S. flexneri* O-antigen	I.p.	Preclinical	-	[83]
	*L. lactis* vector *S. dysenteriae* type-1 OmpA	Oral/I.n.	Preclinical (LacVax)	-	[89]
	*L. lactis* vector *S. flexneri* Intimin-IpaB	Oral	Preclinical	-	[90]

LAVs, live-attenuated vaccines; OMV, outer-membrane vesicle; I.n., intranasal; I.p., intraperitoneal; I.m., intramuscular; C, completed; ID, in development (not yet recruiting); T, terminated; W, withdrawn; IP, in progress (recruiting).

**Table 2:** List of *B. pseudomallei* vaccines.

Vaccine Type	Antigens/Mutant	Immunization Route	Animal Model	Protection	Ref.
**Inactivated**	*Bpm* K96243 (heat-killed)	I.p.	BALB/c mice	80–100% at day 21	[93]
	*Bpm* 576 (heat-killed)	I.p.	BALB/c mice	100% at day 21	[93]
	*Bpm* A2 (Paraformaldehyde-killed)	I.m.	BALB/c mice	50–60% at day 30	[91]
	*Bpm*-liposome	I.n.	BALB/c mice	100% at day 40	[92]
	*B. mallei*	I.n.	BALB/c mice	70% at day 44	[93]
	*B. thailandensis*	I.n.	BALB/c mice	60% at day 44	[93]
**LAVs**	*Bpm* E8 ∆*purN*	I.n.	BALB/c mice	37.5% at day 65	[96]
	*Bpm* E8 ∆*purM*	I.n.	BALB/c mice	100% at day 17	[96]
	*Bpm* 1026b ∆*purM* (Bp82)	S.c.	BALB/c, C57BL/6 mice	60%, 100% at day 60	[97]
	*Bpm* MSHR688 ∆*hisF*	I.p.	BALB/c mice	50% at day 60, 100% at day 21	[98]
	*Bpm* K96243 ∆*aroB*	I.n.	C57BL/6 mice	0% at day 8	[99]
	*Bpm* A2 ∆*aroC*	I.p.	C57BL/6 mice	20–80% up to 5 months	[100]
	*Bpm* 2D2 ∆*ilvI*	I.p.	BALB/c mice	80–100% at day 32	[101]
	*Bpm* 1026b ∆*asd*	I.n.	BALB/c mice	100% at day 16, 0% at day 56	[103]
	*Bm* ATCC 23344 ∆*batA*	I.t.	BALB/c mice	71–100% at day 10, 67–85% at day 55	[104]
	*Bpm* 576 ∆*bipD*	I.p./I.n.	BALB/c mice	60% at day 75	[105]
	*Bpm* K96243 ∆*relA* ∆*spoT*	I.n.	C57BL/6 mice	100% at day 30, 60% at day 55	[106]
	*Bpm* K96243 ∆*tonB* ∆*hcp1*	I.n.	C57BL/6 mice	100% at day 27	[108]
	*Bm* ATCC 23344 ∆*tonB* ∆*hcp1*	I.n.	C57BL/6 mice	87.5% at day 21	[108]
**Subunit**	*Bpm* LolC	I.p.	BALB/c mice	83% at day 42	[111]
	*Bpm* PotF	I.p.	BALB/c mice	50% at day 42	[111]
	*Bpm* Omp3	I.p.	BALB/c mice	50% at day 21	[112]
	*Bpm* Omp7	I.p.	BALB/c mice	50% at day 21	[112]
	*Bpm* Omp85	I.p.	BALB/c mice	70% at day 15	[113]
	*Bpm* OmpW	I.p.	BALB/c, C57BL/6 mice	75% at day 21 and day 80	[114]
	*Bpm* Hcp	I.p.	BALB/c mice	33–80% at day 42	[115]
	*Bm* BimA	I.n.	BALB/c mice	100% at day 21, 20% at day 50	[116]
	*Bm* BopA	I.n.	BALB/c mice	60% at day 50	[116]
**Glycoconjugate**	*Bpm* CPS-LolC	S.c.	BALB/c mice	70% at day35	[122]
	*Bpm* CPS-CRM_197_-Hcp1	S.c.	C57BL/6 mice	100% at day 35	[120]
	*Bpm* CPS-CRM_197_-TssM	S.c.	C57BL/6 mice	80% at day 35	[120]
	*Bpm* TetHc-_SH_CPS	I.p.	BALB/c mice	66.7% at day 35	[121]
	*Bpm* TetHc-LPS	I.p.	BALB/c mice	81% at day 29	[119]
	*Bpm* OPS-AcrA	I.p.	BALB/c mice	40% at day 12	[118]
**OMVs**	*Bpm* 1026b OMV	S.c.	BALB/c mice	60–80% at day 14	[123,124]
	*Bpm* OMV-ODN	S.c.	Rhesus macaques	N.A.	[126]
	*Bpm* 1026b M9 OMV	S.c.	C57BL/6 mice	100% at day 30	[125]
**Reverse** **vaccinology**	AuNP-FlgL-LPS	S.c.	C57BL/6 mice	90% at day 35	[127]
	Combined AuNP- Hemagglutinin-LPS, AuNP-Hcp1-LPS and AuNP-FlgL-LPS	S.c.	C57BL/6 mice	100% at day 35	[127]
	Combined Au-OpcP-LPS and Au-OpcP1-LPS	I.n.	C57BL/6 mice	Up to 90% at day 35	[129]
**Others**	pcDNA3/CpG-*fliC*	I.m.	BALB/c mice	93.9% at day 12	[133]
	pVAX-hTPA-FliC	I.n.	C57BL/6 mice	53% at day 14	[133]
	PIV5- BatA	I.n.	BALB/c mice	80% at day 10, 60% at day 35	[135]

LAVs, live-attenuated vaccines; OMV, outer-membrane vesicle; I.n., intranasal; I.p., intraperitoneal; I.m., intramuscular; I.t., intratracheal; S.c., subcutaneous; N.A., Not Applicable.

## Data Availability

Not applicable.
